# Interplant Communication of Tomato Plants through Underground Common Mycorrhizal Networks

**DOI:** 10.1371/journal.pone.0013324

**Published:** 2010-10-13

**Authors:** Yuan Yuan Song, Ren Sen Zeng, Jian Feng Xu, Jun Li, Xiang Shen, Woldemariam Gebrehiwot Yihdego

**Affiliations:** 1 Key Laboratory of Ecological Agriculture, Ministry of Agriculture, South China Agricultural University, Guangzhou, China; 2 Key Laboratory of Agroecology and Rural Environment of Guangdong Regular Higher Education Institutions, South China Agricultural University, Guangzhou, China; 3 Institute of Tropical and Subtropical Ecology, South China Agricultural University, Guangzhou, China; Agroscope Reckenholz-Tänikon, Research Station ART, Switzerland

## Abstract

Plants can defend themselves to pathogen and herbivore attack by responding to chemical signals that are emitted by attacked plants. It is well established that such signals can be transferred through the air. In theory, plants can also communicate with each other through underground common mycorrhizal networks (CMNs) that interconnect roots of multiple plants. However, until now research focused on plant-to-plant carbon nutrient movement and there is no evidence that defense signals can be exchanged through such mycorrhizal hyphal networks. Here, we show that CMNs mediate plant-plant communication between healthy plants and pathogen-infected tomato plants (*Lycopersicon esculentum* Mill.). After establishment of CMNs with the arbuscular mycorrhizal fungus *Glomus mosseae* between tomato plants, inoculation of ‘donor’ plants with the pathogen *Alternaria solani* led to increases in disease resistance and activities of the putative defensive enzymes, peroxidase, polyphenol oxidase, chitinase, β-1,3-glucanase, phenylalanine ammonia-lyase and lipoxygenase in healthy neighbouring ‘receiver’ plants. The uninfected ‘receiver’ plants also activated six defence-related genes when CMNs connected ‘donor’ plants challenged with *A. solani*. This finding indicates that CMNs may function as a plant-plant underground communication conduit whereby disease resistance and induced defence signals can be transferred between the healthy and pathogen-infected neighbouring plants, suggesting that plants can ‘eavesdrop’ on defence signals from the pathogen-challenged neighbours through CMNs to activate defences before being attacked themselves.

## Introduction

Plants are challenged by a wide variety of pathogens and pests. In response they have developed effective defence systems against these agents based on a combination of constitutive defences as well as induced defences expressed only after an initial signal that invaders are present [1]–[3]. Plants can also establish enhanced defence capacity in plant parts distant from the site of primary attack, thereby providing systematic protection against subsequent invasion [4], [5]. Moreover, many species can increase their defence levels by responding to chemical signals from neighbours that are being attacked by herbivores or pathogens [6]–[13]. When a plant attacked by herbivores emits signals, the neighbouring plants anticipate imminent damage and take timely measure by direct defence; by increasing levels of toxins and repellents [14], or by indirect defence, attracting natural enemies [15], [16]. Although there is increasing evidence of plant-plant communication, the majority of the studies conducted so far have focused on signals transferred from the sender to the receiver by air. Communication via volatile signals, however, is subject to the vagaries of atmospheric conditions.

Mycorrhizae are ubiquitous symbiotic associations between soil-borne fungi and plant roots. Approximate 80% of terrestrial plants establish mutualistic mycorrhizae with arbuscular mycorrhizal fungi (AMF, phylum Glomeromycota), which play a vital role in soil fertility and plant nutrition [17]. Mycorrhizae enhance host plant defence against many soil-borne fungal pathogens [17]–[19]. Mycorrhiza increased tomato resistance not only to soil borne disease caused by *Phytophthora nicotianae* var. parasitica [20], but also to foliar disease caused by necrotrophic fungus *Alternaria solani* [21]. Mycorrhizal symbiosis is a key factor in the below ground network essential for functioning of territorial ecosystems [22]. Mycorrhizal fungal diversity determines plant biodiversity, ecosystem variability and productivity [23].

Mycorrhizal fungal mycelia can extend from one plant's roots to another to form common mycorrhizal networks (CMNs) due to lack of specificity of arbuscular mycorrhiza [24], [25]. CMNs can also be established via anastomoses by which different branches of the same or different hyphae fuse to constitute a mycelial network [26]–[28]. Different plants and even different species can be interconnected through CMNs. A single individual mycelium of a widely distributed unidentified *Glomus* species in undisturbed coastal grassland could cover an area that is at least 10 m in length [29]. Nutrients such as nitrogen and phosphorus and other elements may then move from plant to plant via CMNs [27], [30], [31,]. Nitrogen fixed by legume plants can be transferred to associated non-N_2_-fixing crops [30], [32]. Movement of water through CMNs is potentially important to plant survival during drought [33]. Such nutrient transfer between plants connected by CMNs is bidirectional [34]. CMNs have the potential to influence patterns of seedling establishment, interplant competition, plant diversity, and plant community dynamics [25], [35], [36]. CMNs appear to facilitate seedling establishment through rapid fungal inoculation as well as transfer of carbon, nutrients, or water from neighboring residual trees [35].

The existence of these connections raises possibility that the CMNs may serve as a channel for information exchange between the connected plants [36]. However, it is so far unknown whether defence signals may transfer from one plant to the other through CMNs. We conducted this study to assess whether defence signals could be transferred from tomato plants (*Lycopersicon esculentum* Mill.) challenged by *Alternaria solani* Sorauer to neighbouring healthy tomato plants connected by common mycorrhizal mycelia of *Glomus mosseae*.

## Results

**Figure 1:**
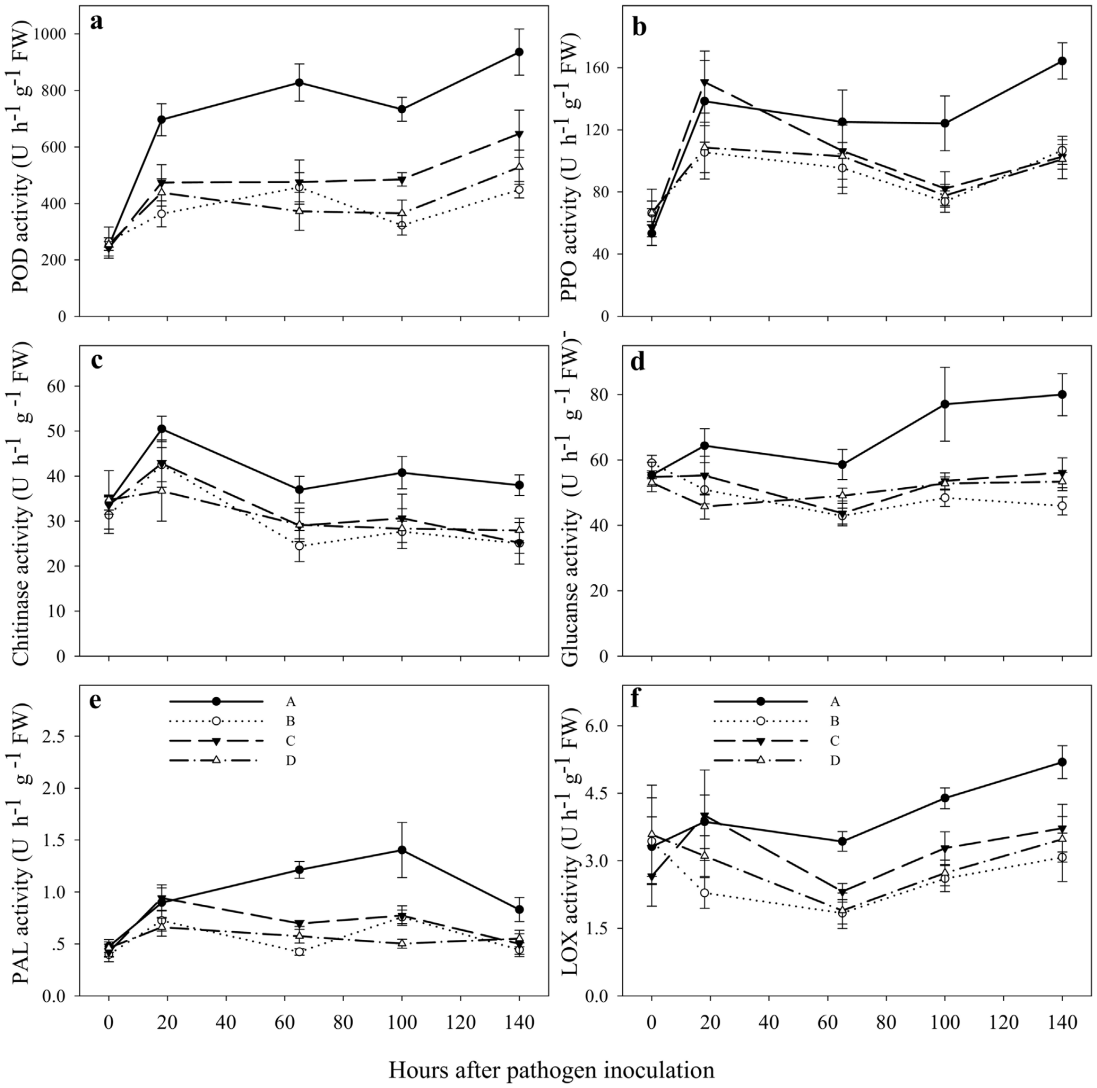
Levels of six defence-related enzymes in leaves of tomato ‘receiver’ plants in response to common mycorrhizal networks (CMNs) connected with *Alternaria solani*-infected neighbouring tomato. *Glomus mosseae* was used to established the CMNs. Six defence-related enzymes are peroxidase (POD), polyphenol oxidase (PPO), chitinase, β-1,3-glucanase, phenylalanine ammonia-lyase (PAL) and lipoxygenase (LOX). Four treatments included: **A**) a healthy tomato ‘receiver’ plant was connected with a neighboring *A. solani*-challenged tomato ‘donor’ plant through CMNs; **B**) a healthy ‘receiver’ plant was grown near *A. solani*-challenged ‘donor’ plant but no mycorrhiza was applied; **C**) a healthy mycorrhizal ‘receiver’ plant was grown near the pathogen-challenged mycorrhizal ‘donor’ plant but the two tomato plants separated by a water-proof membrane and **D**) a healthy ‘receiver’ plant was connected with the neighbouring plant by CMNs without pathogen inoculation. Values are means ± standard error from three sets of independent experiments with three pots per treatment for each set of experiments. Significant differences among treatments were tested at *P* = 0.05 by Tukey post-hoc test (**Supporting Information Table S3**).

Six defence-related enzymes, including peroxidase (POD), polyphenol oxidase (PPO), chitinase, β-1,3-glucanase, phenylalanine ammonia-lyase (PAL) and lipoxygenase (LOX), in the leaves of neighbouring ‘receiver’ plant were analyzed. The activity of POD was significantly higher in healthy tomato plants that were connected by the *G. mosseae* CMNs with the pathogen-challenged tomato plants (Fig 1a). The POD activity in ‘receiver’ plants of treatment A was, on average, higher by 81.0, 74.1 and 122.6% than that of treatment B, C and D, respectively at 65 h after pathogen inoculation of ‘donor’ plants. In contrast, the difference in POD activity in treatments B, C and D were less variable. The enzymatic activity of PPO in ‘receiver’ tomato plants in treatment A was significantly higher at 65, 100 and 140 h after pathogen inoculation than PPO activity in treatment B, C and D (Fig 1b). PPO activity in treatment A increased by 68.2, 51.1 and 59.9% at 100 h after pathogen inoculation, and increased 53.8, 60.1 and 62.3% at 140 h after pathogen inoculation compared with that in treatment B, C and D, respectively. In the other treatment conditions (B, C and D), however, due to the absence of a CMN, the activity of PPO was not significantly different. Upon pathogen challenge in ‘donor’ plants, chitinase activity in the healthy ‘receiver’ plants in treatment A was significantly higher 65 h after the pathogen inoculation (Fig 1c). The chitinase activity displayed increases of 51.6, 27.6 and 27.6%, respectively in the healthy ‘receiver’ plants of treatment A compared to those in treatment B, C and D at 65 h after pathogen inoculation.

The activity of β-1,3-glucanase increased with 59.3, 43.8 and 46.0%, respectively relative to those in treatments B, C and D at 100 h after the pathogen inoculation (Fig 1d). The PAL activity increased in the healthy ‘receiver’ plants of treatment A 65 h after the pathogen inoculation and reached maximum at 100 h, declining thereafter (Fig 1e). The activity of PAL in treatment **A** was, on average, higher by 84.2, 81.8 and 180%, respectively than those in treatments B, C and D at 100 h after the pathogen inoculation. In contrast, PAL and β-1,3-glucanase activities were not significantly different in the ‘receiver’ plants in treatments B, C and D with neither mycorrhizal inoculation, common mycorrhizal network nor pathogen challenge. The LOX activity in the healthy ‘receiver’ plants of treatment A was also significantly higher than that in treatments B, C and D at 65 h after the pathogen inoculation and thereafter (Fig 1f). Although mycorrhization in the healthy ‘receiver’ plants led to some increase in LOX in treatment C and D, LOX induction was more pronounced in the presence of CMNs connection with pathogen-challenged neighbours.

**Figure 2:**
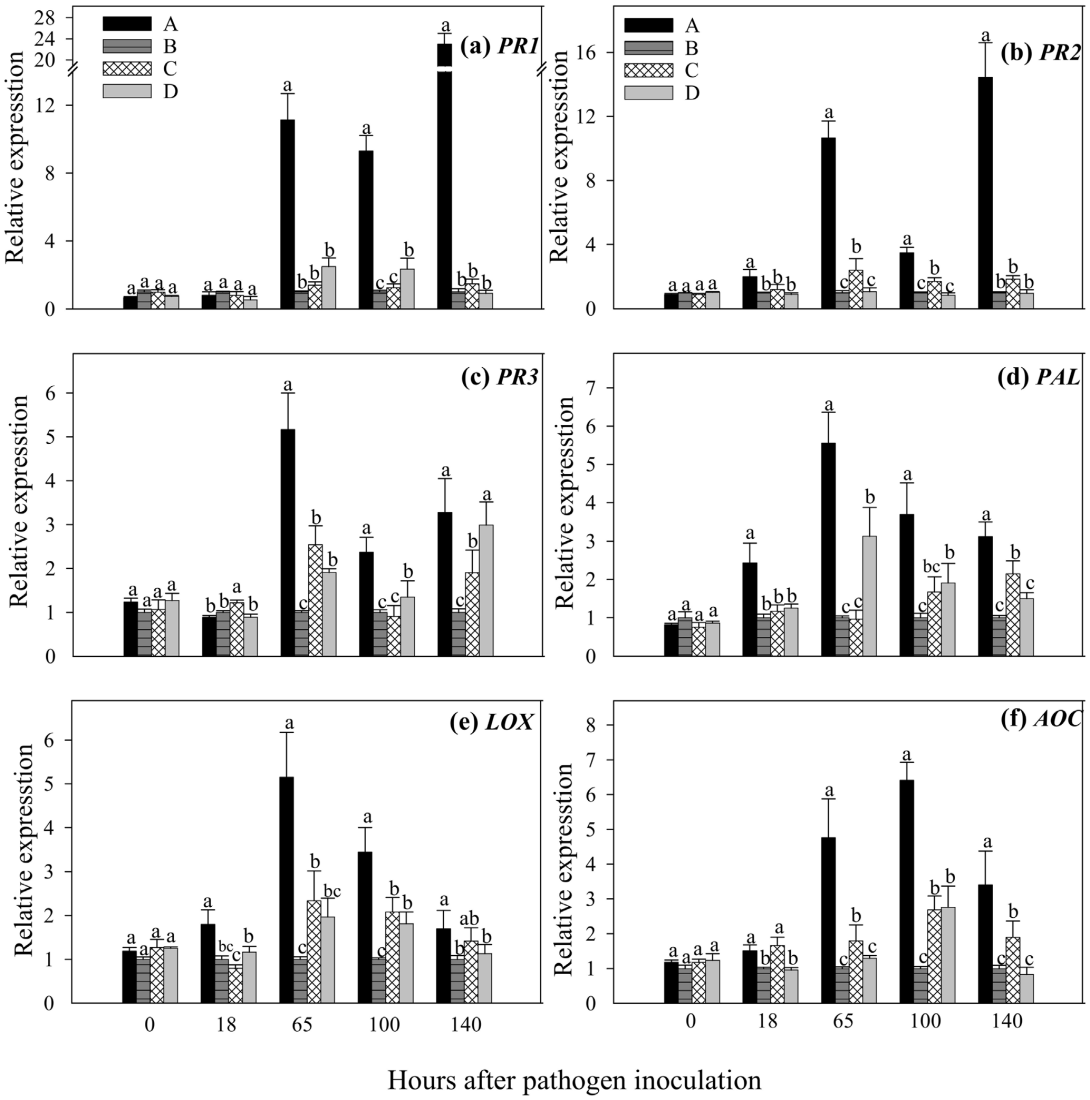
Expression of six defence-related genes in leaves of tomato ‘receiver’ plants in response to common mycorrhizal networks (CMNs) connected with *Alternaria solani*-infected neighbouring tomato. *Glomus mosseae* was used to established the CMNs. Quantitative real time RT-PCR was used to detect the transcripts of six defence genes encoding the pathogen-related proteins (*PR1*), basic type *PR-2* (β-1,3-glucanase) and *PR-3* (chitinase), phenylalanine ammonia-lyase (*PAL*), lipoxygenase (*LOX*) and allene oxide cyclase (*AOC*). **A**) a healthy tomato ‘receiver’ plant was connected with a neighboring *A. solani* challenged tomato ‘donor’ plant through CMNs; **B**) a healthy ‘receiver’ plant was grown near *A. solani*-challenged ‘donor’ plant but no mycorrhiza was applied; **C**) a healthy mycorrhizal ‘receiver’ plant was grown near the pathogen-challenged mycorrhizal ‘donor’ plant but the two tomato plants separated by a water proof membrane and **D**) a healthy ‘receiver’ plant was connected with the neighbouring plant by CMNs without pathogen inoculation. Values are means + standard error from three sets of independent experiments with three pots per treatment for each set of experiments. Significant differences (*P*<0.05 using Tukey post-hoc test) among treatments in a group are indicated by different letters above bars.

In the ‘receiver’ plant leaves, we used quantitative real time RT-PCR to detect the transcripts of six defence genes: genes encoding the pathogen-related proteins (*PR1*), basic type *PR-2* (β-1,3-glucanase) and *PR-3* (chitinase); phenylalanine ammonia-lyase (*PAL*) in the phenylpropanoid pathway; *LOX* and allene oxide cyclase (*AOC*), which are two key enzymes of the jasmonic acid biosynthesis pathway. The ‘receiver’ tomato plants greatly increased the expression of all six defence-related genes when connected with pathogen-challenged neighbouring plants via CMNs (Fig 2). *PR1* transcripts in the ‘receiver’ plants in treatment A were upregulated 11.1, 9.3 and 23.0-fold at 65, 100 and 140 h after pathogen inoculation, respectively in comparison to those in treatment B, which did not have CMN connections with pathogen-challenged neighbouring plants (Fig 2a). *PR2* and *PR3* showed an approximate 14.5 and 3.3-fold increase of expression in the ‘receiver’ plants in treatment A compared with those of treatment B, respectively, at 140 h after pathogen inoculation (Fig 2b). The expression levels of *PAL*, *LOX* and *AOC* increased 5.6, 5.2 and 4.8-fold at 65 h, 3.7, 3.4 and 6.4-fold at 100 h, and 3.1, 1.7 and 3.4-fold at 140 h after pathogen inoculation, respectively (Fig 2d, e, f). The gene expression levels in the ‘receiver’ plants in treatment C and D increased to some extent, which may have resulted from root infection by the mycorrhizal fungus, but they were not as high as those in treatment A in which those ‘receiver’ and “donor’ plants were linked by CMNs.

However, no significant difference was found in enzymatic activities and gene expression of ‘receiver’ plants among four treatments at the beginning of *A. solani* inoculation (T = 0 h) on ‘donor’ plants (Fig 1 and 2).

To test whether this induced defence by CMN interplant communication can enhance tomato resistance to early blight disease, ‘receiver’ plants in four treatments were inoculated with *A. solani* after CMNs had been established and 65 h after ‘donor plant’ had been inoculated with *A. solani*. Although mycorrhizal formation enhanced tomato resistance to leaf disease by *A. solani*, consistent with the other findings [21], pathogen-challenged neighbouring plants further increased plant resistance to early blight when they were connected by CMNs (Table 1). Disease incidence and severity were significantly reduced in ‘receiver’ plants connected by CMNs with the pathogen-challenged tomato plants (treatment A) compared with the control plants in treatment B that have no CMN connection with the neighbouring pathogen-challenged tomato plants (Table 1). Disease incidence and index of ‘receiver’ plants in treatment A decreased by 50.2 and 63.8%, respectively compared with those in treatment B, which had no mycorrhizal connection with the pathogen-challenged ‘donor’ plants. Although ‘receiver’ plants in treatment A and D had similar mycorrhizal colonization and close AM infection rates (Table 1), disease resistance in ‘receiver’ plants in treatment A was significantly higher than that in treatment D.

**Table 1:** Mycorrhizal infection rates, disease incidences and indices of tomato ‘receiver’ and ‘donor’ plants infected by *Alternaria solani*.

Treatment	‘Receiver’ plants			‘Donor’ plants		
	Disease incidence (%)	Disease index (%)	Mycorrhizal infection (%)	Disease incidence (%)	Disease index (%)	Mycorrhizal infection (%)
A	31.5±2.3 c	16.6±1.2 c	36.2±2.8 b	32.9±3.4 c	14.7±1.2 c	55.5±1.5 b
B	63.3±2.5 a	45.8±2.6 a	0 c	71.9±3.1 a	53.9±4.5 a	0 d
C	48.1±3.3 b	28.5±2.5 b	48.7±0.5 a	47.7±3.1 b	25.9±1.3 b	42.7±0.9 c
D	52.4±2.6 b	28.0±1.5 b	36.1±1.1 b	0 d	0 d	60.3±1.7 a

‘Receiver’ plants of all four treatments were inoculated with *A. solani* 65 h after pathogen inoculation in the ‘donor’ plants. Four treatments included: A) a healthy tomato ‘receiver’ plant was connected with a neighboring *A. solani* challenged tomato ‘donor’ plant through common mycorrhizal network of *Glomus mosseae*; B) a healthy ‘receiver’ plant was grown near *A. solani* challenged ‘donor’ plant but no mycorrhiza was applied; C) a healthy mycorrhizal ‘receiver’ plant was grown near the pathogen challenged mycorrhizal ‘donor’ plant but the two tomato plants separated by water proof membrane and D) a healthy ‘receiver’ plant was connected with the neighboring plant by common mycorrhizal network without pathogen inoculation. Four sets of bioassays were independently carried out and three pots per treatment were set up for each set of bioassays. Values are means ± standard error. Significant differences (*P*<0.05 using Tukey post-hoc test) among treatments in the same column are indicated by different letters. Results of ANOVA analysis and each set of bioassays are presented in the **Supporting Information (Table S1 and Table S2)**.

## Discussion

We demonstrate that CMNs can serve as underground communication conduit transferring defence signals and disease resistance between healthy and pathogen-infected neighbouring plants. Induced defence in neighbouring plants can be explained by two possible mechanisms: i) aboveground communication can take place by volatiles [11], [37]–[40] and ii) belowground communication can be mediated by root exudates [10], [41]. In this study, we excluded volatile communication by covering the infected ‘donor’ plant with an air-tight plastic bag after pathogen inoculation. We also excluded the belowground communication by root exudates in treatment B by growing tomato plants without mycorrhiza but the neighbouring ‘donor’ plants being pathogen-infected. If root exudates mediate plant-plant communication, induced defence responses could be detected in ‘receiver’ plants in treatment B. We found the highest disease incidence and severity of ‘receiver’ plants in treatment B among the four treatments (Table 1), suggesting that no induced defence occurred when the two plants were not connected with CMNs.

We eliminated the possible mycorrhization effects on enhanced defence by treatment C and D. Both ‘donor’ and ‘receiver’ plants in treatment C were inoculated by the mycorrhizal fungus *G. mosseae*, but they were separated by a water-proof membrane (Fig 1a). No CMN was established between the ‘donor’ and ‘receiver’ plants (Table 1). We found significantly lower levels of defence-related enzymatic activities, gene expression and disease resistance in the leaves of ‘receiver’ plants in treatment C than those in treatment A, although both neighbouring ‘donor’ plants in treatment A and C were inoculated with the pathogen. The difference between treatment A and D was that the ‘receiver’ plants in treatment A were connected by CMNs with pathogen-challenged ‘donor’ plants, while these in treatment D were connected with non-pathogen-challenged ‘donor’ plants. Significantly higher levels of defence related enzymatic activities and gene transcripts in the ‘receiver’ plants of treatment A compared to treatment D suggest that defence signals have been transferred from pathogen-challenged ‘donor’ plants to healthy ‘receiver’ plants in treatment A. No significant difference among treatments was found in enzymatic activities and gene expression in the ‘receiver’ plants before ‘donor’ plants received pathogenic inoculation [42]. Further experiments with plants grown under axenic conditions should be performed to exclude possible mycorrhizal effects on soil microflora multiplication and to confirm our findings under more controlled experimental conditions. It is also important to determine the expression pattern of those house-keeping genes, which are not expected to be affected by pathogen infection, when investigating the expression levels of defense genes in response to communication with pathogen-challenged neighbours through CMNs. Such genes should have the same expression levels in all treatments and should not be affected by the treatments with or without mycorrhizal networks.

In plants, recognition of a potential invader through the detection of signal molecules is a requirement to initiate an effective defence response [43]. However, our tested tomato plants were neither challenged by a pathogen nor contacted with volatile signals from ‘donor’ plants. The only possible communication between the healthy ‘receiver’ plants and pathogen-challenged ‘donor’ plants was through CMNs. We suggest that as plants exchange nutrients and water through CMNs, defence signals can also be transferred from the pathogen-challenged plants to the neighbouring healthy plants via CMNs. Healthy plants by recognizing these signals, could anticipate the likely attack and induce their defence responses. In this study mycorrhizal ‘donor’ plants are more likely to produce the defence signals upon pathogen infection, and these signals are then transferred from ‘donor’ plants to neighboring ‘receiver’ plants through CMNs. The ‘receiver’ plants may “eavesdrop” on the signals and then activate their defence responses. Eighteen hours after pathogen inoculation on the leaves of ‘donor’ plants three genes including *PR2*, as well as LOX in jasmonic acid (JA) pathway and PAL salicylic acid (SA) pathway, were up-regulated in the leaves of ‘receiver’ plants (Fig 2b, d, e), suggesting that at this time point the ‘receiver’ plants had already triggered their defence responses. Since it took time to get infection on ‘donor’ plants and then activate defence responses in ‘donor’ plants, it would be much less than 18 h to transfer defence signals from ‘donor’ to ‘receiver’ plants. It was found that a mass of particles (e.g., vacuoles, mitochondria, nuclei, and fat droplets) moved at the speed of 1.8 µm/s (approximately 15.5 cm/d) in both directions within the hyphal bridges of *Glomus caledonium* [44]. It is more likely that the defence signal compounds move faster in hyphal networks compared with cell organelles since the signal molecules are much smaller and easier to be transferred [45].

Induction of both SA and JA pathways suggests that the possible signals from ‘donor’ plants to ‘receiver’ plants are SA and JA (Fig 1 and 2). Methyl salicylate has been suggested as a crucial long distance SAR (systemic acquired resistance) signal in tobacco [45]. JA also plays a central role in induced systemic resistance and in plant interactions with resistance-inducing beneficial microbes [46], [47]. Other signals may also be transferred from ‘donor’ plants to neighboring ‘receiver’ plants through CMNs [48]. Further study is required to determine the exact signal compounds transferring through AMF networks.

Infection by necrotizing pathogens or beneficial microbes may provoke some plants to develop a unique physiological state called “priming” [49]–[51]. Primed plants display faster and/or stronger activation of various cellular defence responses after pathogen and insect attack [52]. Priming is an important mechanism in mycorrhiza-induced resistance [53]. Colonization of tomato roots by *G. mossae* systemically protects the plant against infection by *Phytophthora parasitica* through priming [54]. Our study showed that mycorrhizal inoculation itself did not affect most enzyme activities tested (Fig 1) and only had marginal effects on transcripts of defence-related genes (Fig 2). However, pre-inoculation of tomato with AMF primed defence responses in ‘donor’ plants after pathogen attack [42]. In our case induction of defence responses in pre-inoculated plants was much higher and quicker than that in non-inoculated plants upon *A. solani* infection. All mycorrhizal tomato plants increased their resistance to early blight disease by *A. solani* (Table 1). Although the receiver plants used for the gene expression and enzyme analysis were not challenged by the pathogen, they got defence signals from the infected ‘donor’ plant and induced the gene expression and enzymatic activity.

Based on these results, that plants connected with pathogen-infected neighbours by CMNs had less disease damage, higher levels of defence-related enzymatic activities and gene expression than controls without infected neighbours or without CMNs, we suggest that tomato plants can ‘eavesdrop’ on defence signals from the pathogen-challenged neighbours through CMNs to activate defence responses and increase their disease resistance against potential pathogen. This discovery extends the possible functional roles of mycorrhizas and CMNs, namely with additional protection against diseases through CMNs formation. In other words, the CMNs not only may function as nutrient and water allocation networks [35], but also could act as defence networks in plant communities.

Volatile-mediated plant-plant communication is well documented [7], [55]. However, distribution of the volatile signal molecules depends on distance between the plants [39], variability of wind direction and speed. The CMNs plants can overcome these uncertainties of signal transfer by having physical connections. One can argue by extension that the CMNs may induce systemic defence in ecosystems, minimizing disease occurrence and severity in plant communities. Since approximate 80% land plants are connected with mycorrhizal fungi we argue that the CMNs communication is evolutionarily more advanced for its reliability and efficiency in signal transfer than airborne communication by volatiles. This enhanced reliability and efficiency of signal transfer in CMNs can be argued as corollary to TV signal transfers between the cable and airborne systems.

To our knowledge, this study is the first to show that CMNs may function as a defence communication conduit between infected and healthy plants. Further studies on plant-plant communication will promote our understanding of systemic defence in natural ecosystems, which in turn may provide clues to manipulate plant defences in agroecosystems.

## Materials and Methods

### Plant and fungal materials

Tomato seeds (*Lycopersicon esculentum*, Mill. cv. Jin Bao) were surface-sterilized with 10% H_2_O_2_ and rinsed five times with sterile distilled water before sowing in autoclaved quartz sand. After 10 d the seedlings were utilized for the experiment.

The starting inocula of mycorrhizal fungus *Glomus mosseae* (Nicol. & Gerd) Gerdemann & Trappe BEG 167 used in this experiment, were kindly provided by Prof. Runjin Liu at Qingtao Agricultural University. The mycorrhizal inoculum was produced in pot culture using corn (*Zea mays* L.) plants and autoclaved sand media [56]. A mixture of rhizospheric sand containing spores, mycelium and colonized plant roots from trap cultures (*Z. mays*) with 35 infective propagules per gram was used for mycorrhizal inoculation.

The pathogen (*Alternaria solani* Sorauer ACCC36110) was kindly provided by Prof. Erxun Zhou of Department of Plant Pathology at South China Agricultural University. The fungus was cultivated for 6 d on potato dextrose broth, amended with 100 mg/l streptomycin sulfate, at 28°C in darkness, on a shaker at 150 rpm. After the incubation period, the fungal culture was centrifuged at 1000 *g*, re-suspended in sterilized water, and re-centrifuged. The spore concentration was determined and adjusted to 10^6^ conidia/ml using a hemacytometer. To avoid contamination, the ‘receiver’ plant was covered with air-tight plastic bag during application of the pathogen.

### Chemicals

TRIzol reagent, M-MLV reverse transcriptase, Taq polymerase, RNase inhibitor and dNTPs were purchased from TaKaRa (Shuzo Co. Ltd., Shiga, Japan), while MOPS and DEPC were purchased from AMRESCO (Solon, OH).

### Experimental design

**Figure 3:**
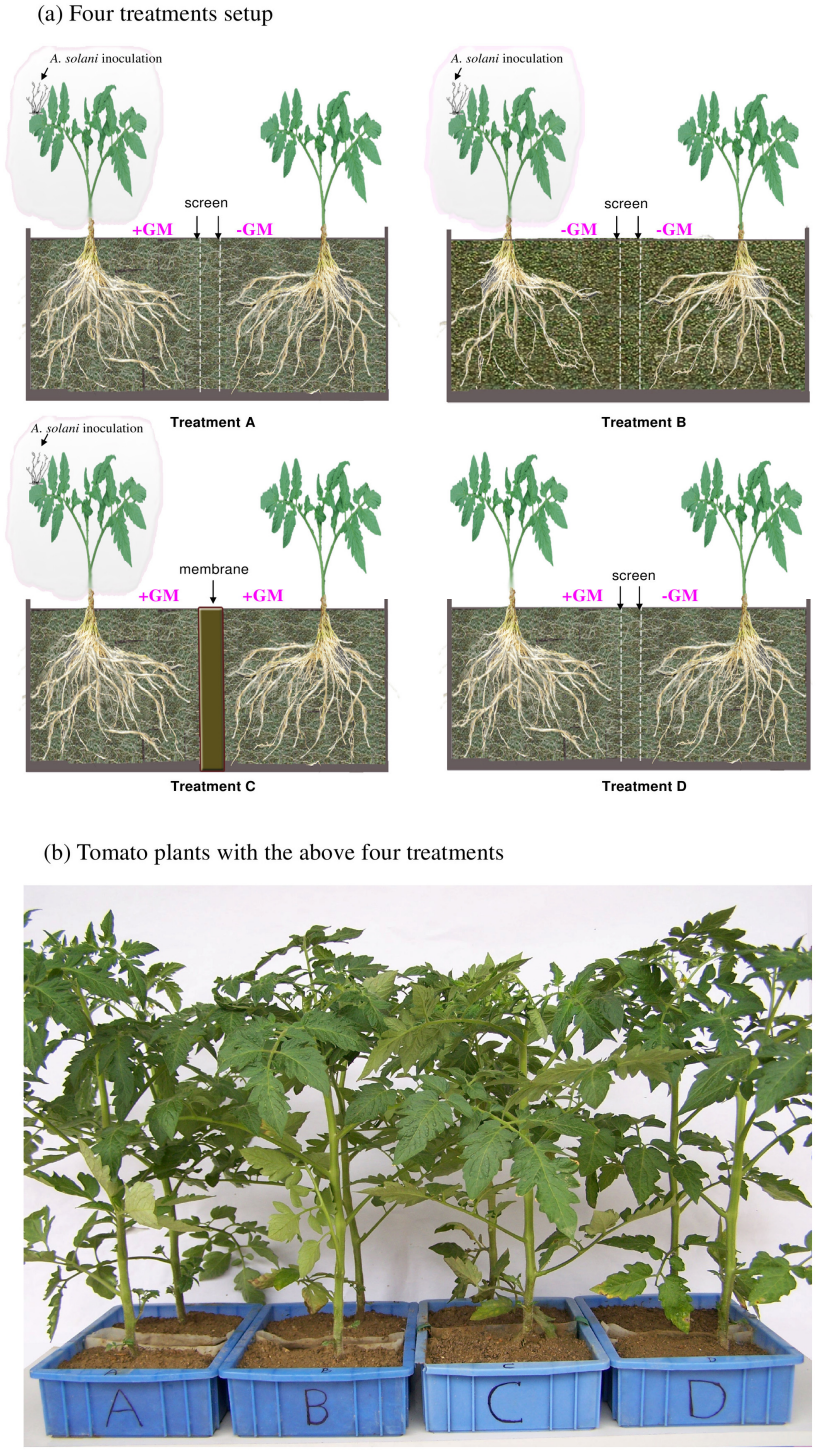
Experimental design. (a) Four treatments included: **A**) a healthy tomato ‘receiver’ plant was connected with a neighboring *Alternaria solani*-challenged tomato ‘donor’ plant through common mycorrhizal networks (CMNs) of *Glomus mosseae* (GM); **B**) a healthy ‘receiver’ plant was grown near *A. solani*-challenged ‘donor’ plant but no mycorrhiza was applied; **C**) a healthy mycorrhizal ‘receiver’ plant was grown near the pathogen-challenged mycorrhizal ‘donor’ plant but the two tomato plants separated by a water-proof membrane and **D**) a healthy ‘receiver’ plant was connected with the neighbouring plant by CMNs without pathogen inoculation. (b) Tomato plants with the four treatments. In Figure 3a +GM refers to inoculation with *G. mosseae* in the compartment, and -GM refers to non-inoculation in the compartment. White fine filamentous networks refer to hyphal networks of *G. mosseae*. The hyphal networks across the two fine stainless steel screens in treatment A and D indicate the establishment of CMNs between ‘donor’ and ‘receiver’ plants.

*G. mosseae* was used to establish CMNs between tomato plants, and *A. solani*, causal agent of tomato early blight disease, was used for pathogenic inoculation. Two tomato plants were grown in a rectangular pot measuring 29×13×11 cm (length×height×width) and separated by two fine stainless steel screens (25 µm, TWP Inc. Berkeley, CA, USA), which divided each pot into two equal compartments (Compartment Ι and ΙΙ), to prevent direct root contact but allow mycelia of mycorrhizal fungus to get through to establish common mycorrhizal networks (CMNs) between the two tomato plants in the same pot. Four treatments (A, B, C and D) were designed to determine effects of CMNs and to exclude possible effects of root exduates and mycorrhization (Fig 3). A) A healthy ‘receiver’ plant was connected with a neighbouring *A. solani*-challenged ‘donor’ plant through a *G. mosseae* CMN; B) a healthy ‘receiver’ plant was grown near *A. solani*-challenged ‘donor’ plant but no mycorrhizal fungus was applied; C) a healthy mycorrhizal ‘receiver’ plant was grown near the pathogen-challenged mycorrhizal ‘donor’ plant but the two tomato plants were separated by a water-proof membrane to prevent any root and mycelial contact between the two compartments and D) a healthy ‘receiver’ plant was connected with the neighbouring plant by a CMN without pathogen inoculation.

To prevent direct root contact of plants in both compartments, two screens (in treatments A, B and D) were separated by 3 cm gap filled with sterile sands. Each compartment was filled with 1.5 kg sterilized sieved field soil/sand mixture (2∶1). The brown loam soil was collected from the university campus in Guangzhou (China) containing 2.49% organic matter, 0.119% total N, 55.37 mg/kg available P with a pH of 5.51. The soil/sand mixture was sterilized by autoclaving. Compartment Ι in all pots of treatments A, B and D for enzymatic and molecular analysis experiment did not receive any inoculation and contained only one healthy tomato plant which was denoted ‘receiver’ plant. However, the tomato plants in Compartment ΙΙ which were denoted as ‘donor’ plants received (1) inoculation of both AMF *G. mosseae* and pathogen (*Alternaria solani* Sorauer) in treatment A; (2) only *A. solani* inoculation in treatment B; (3) inoculation of both AMF *G. mosseae* and *A. solani* in treatment C, and (4) only *G. mosseae* inoculation in treatment D. For *G. mosseae* inoculation 100 g of the sand substrate containing the inocula of *G. mosseae* was applied to the Compartment ΙΙ in treatments A and D before sowing. In treatments C each compartment (I and ΙΙ) received 50 g of sand inocula. The ‘receiver’ plant and ‘donor’ plant’ were connected by common mycorrhizal networks in treatment A, but not connected in treatment B because there was no mycorrhizal inoculation in compartment ΙΙ. In treatment C, although plants in both compartment I and II were inoculated with *G. mosseae* and plants in compartment II were inoculated with *A. solani*, there was no CMN connection because they were separated by a water-proof membrane. In treatment B the sands (100 g) mixing with sterile soil for growth media were obtained from the growth media of corn without mycorrhizal inoculation.

Two 10-day-old tomato seedlings were sown in each compartment. The seedlings were thinned to one plant per compartment 7 d after planting. Plants were grown in a growth chamber at 25±1°C with a 16 h photoperiod, 150 Md/m^2^/s PAR and 60% relative humidity. Seedlings were watered daily and fertilized every 7 d with 50 ml of nutrient solution (5 ml 1 M KNO_3_, 5 ml 1 M Ca(NO_3_)_2_, 1 ml 1 M MgSO_4_, 2 ml 1 M KH_2_PO_4_, 1 ml H_3_BO_3_, 1 ml MnCl_2_, 1 ml ZnSO_4_, 1 ml CuSO_4_ and 1 ml FeEDTA in one liter solution) per compartment. Pots were randomized in the growth chamber and re-randomized every 10 days during the growing period.

After the common mycorrhizal network had established, tomato ‘donor’ plants were inoculated with the pathogen *Alternaria solani*, the causal agent of tomato early blight disease. Based on the experience gained during the preliminary experiments, the common mycorrhizal network was established 35 d after transplanting and the mycorrhizal infection rates of ‘receiver’ plants in treatments A, C and D were 42.3, 64.7 and 46.7%, respectively. Therefore, forty days after planting, tomato leaves of ‘donor’ plants in Compartment ΙΙ in treatments A, B and C were inoculated by carefully spraying with a 10^6^ conidia/ml suspension of *A. solani*. Thirty milliliters of the conidia suspension were applied to each plant. To ensure the high relative humidity needed during spore germination, as well as to eliminate possible volatile signal contact between ‘receiver’ and ‘donor’ plants, all ‘receiver’ plants were covered with an air-tight plastic bag during pathogen inoculation, and all inoculated and control ‘donor’ plant was covered with an air-tight plastic bag after pathogen inoculation.

Leaves of ‘receiver’ plants in Compartment Ι were harvested 0, 18, 65, 100 and 140 hours after pathogen inoculation for real-time RT-PCR and enzymatic analysis. The tomato roots were examined for the establishment of mycorrhizal networks through the micropores of the mesh at harvest to avoid wound stress on the healthy tomato plants. Fifty 1 cm root samples were taken from each tomato plant, cleaned and stained to measure AM colonization [56].

### Bioassay

To test whether induced defence by CMNs communication can enhance disease resistance, a bioassay was conducted to compare the disease incidence and index of both ‘receiver’ and ‘donor’ plants. In all four treatments, CMN establishment and pathogen inoculation in the ‘donor’ plants were the same as those in the enzymatic assay experiment. However, in bioassay experiment ‘receiver’ plants in Compartment Ι of all four treatments were inoculated with *A. solani* 65 h after pathogen inoculation in the ‘donor’ plants. The disease incidence and index of ‘receiver’ plants were recorded 7 d after pathogen inoculation. Disease incidence was defined as percentage of diseased leaves. Disease severity was estimated using Disease Index (*DI*) calculated from disease grades 0–5 [57] using the formula:
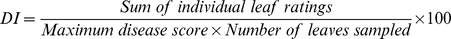
Four sets of bioassays were independently carried out and three pots per treatment were set up for each set of bioassays.

### Enzyme Assays

Induction of defence enzymes has been correlated with defence against pathogen invasion in tomato [58]. All six enzymes tested here are involved in plant defence response to pathogens. These oxidative enzymes include peroxidase (POD) and polyphenol oxidase (PPO), which catalyse the formation of lignin and other oxidative phenols. Plant POD has been reported to catalyze the last steps in the biosynthesis of lignin and hydrogen peroxide. Phenylalanine-ammonia-lyase (PAL) is involved in phytoalexin or phenolic compound biosynthesis. Lipoxygenase (LOX) catalyses the initial reaction in jasmonic acid biosynthesis pathway, which inserts molecular oxygen into position 13 of *α*-linolenic acid. Hydrolytic enzymes include pathogenesis-related protein 1 (PR1), β-1,3-glucanases (PR-2 family) and chitinases (PR-3 family), which degrade the fungal cell wall and cause lysis of fungal cell.

Leaf samples (0.2 g) were harvested from the healthy tomato plant in compartment Ι in all treatment conditions (A, B, C, and D). Leaves were ground in liquid nitrogen and homogenized in 2.0 ml ice cold 0.05 M phosphate buffer (pH 7.2 for POD, pH 7.8 for PPO) containing 1% (w/v) polyvinylpyrrolidone (PVP). The homogenate was centrifuged at 12000 *g* for 15 min at 4°C. The supernatant was collected and used for assaying the activities of peroxidase (POD) and polyphenol oxidase (PPO) by using spectrophotometer. POD activity was determined as described [59]. PPO activity was assayed with 0.05 M catechol as a substrate by a spectrophotometric procedure [60]. Leaf samples (0.1 g) were ground in liquid nitrogen and extracted with 2 ml 0.05 M sodium acetate buffer (pH 5.0) and centrifuged at 12 000 *g* for 15 min at 4°C. The supernatant was used for the enzyme assay of β-1,3-glucanase and chitinase. β-1,3-Glucanase activity was assayed by the laminarindinitrosalicylic acid method [61]. The chitinase activity was assayed as described [62].

Phenylalanine ammonia-lyase (PAL) activity was determined as the rate of the conversion of L-phenylalanine to trans-cinnamic acid at 290 nm. Leaf samples (0.2 g) were ground using liquid nitrogen and homogenized in 1 ml ice cold 0.05 M sulphate buffer, pH 8.8 containing 7 mM 2-mercaptoethanol and 0.1 g insoluble polyvinylpyrrolidone. The homogenate was centrifuged at 12000 *g* for 20 min. The supernatant was used as enzyme analysis. PAL activity was determined spectrophotometrically [63].

Lipoxygenase (LOX) activity was measured as conjugated diene formation [64]. Leaf samples (0.2 g) were ground using liquid nitrogen and extracted with 1 ml ice-cold 0.5 M TRIS-HCl buffer (pH 7.6) and centrifuged at 12 000 *g* for 15 min at 4°C. The supernatant was kept at 4°C until used. The substrate contained 1.6 mM linoleic acid and 0.5% (v/v) Tween 20 in 0.1 M phosphate buffer (pH 7.6). The reaction was initiated by the addition of 0.2 ml crude extract in 4.8 ml of the substrate. Diene formation was followed as increase of absorbance at 234 nm.

### Real-time RT-PCR analysis

Differential expression of selected genes was verified by real-time RT-PCR using the RNA samples isolated from tomato leaves obtained from the four treatments. The *Ubi3* gene was used as a reference gene. Total RNA from tomato leaves was extracted and isolated according to the method of Kiefer *et al.* [65] including a DNase (Promega, Madison, USA) treatment. First strand cDNA was synthesized from 1 µg of total RNA using ImProm-II™ Reverse transcription system (Promega, Madison, USA) according to the manufacturer's instructions.

The primers for target's genes *PR1*, *PR2*, *PR3*, *PAL*, *LOX* and *AOC* were designed by Primer 3.0 software (Applied Biosystems, http://fokker.wi.mit.edu/primer3/input.htm) based on tomato mRNA sequences deposited in GenBank. We used the following primers: *PAL* sense, 5′-CTGGGGAAGCTTTTCAGAATC-3′, and antisense, 5′-TGCTGCAAGTTACAAATCCAGAG-3′; *LOX* sense, 5′-ATCTCCCAAGTGAAACACCACA-3′, and antisense, 5′-TCATAAACCCTGTCCCATTCTTC-3′; *AOC* sense, 5′-CTCGGAGATCTTGTCCCCTTT-3′, and antisense, 5′-CTCCTTTCTTCTCTTCTTCGTGCT-3′; *PR1* sense, 5′-GCCAAGCTATAACTACGCTACCAAC-3′, and antisense, 5′-GCAAGAAATGAACCACCATCC-3′; *PR2* sense, 5′-GGACACCCTTCCGCTACTCTT-3′, and antisense, 5′-TGTTCCTGCCCCTCCTTTC-3′; *PR3* sense, 5′-AACTATGGGCCATGTGGAAGA-3′, and antisense, 5′-GGCTTTGGGGATTGAGGAG-3′; *Ubi3* (Internal standard) sense, 5′-TCCATCTCGTGCTCCGTCT-3′, and antisense, 5′-GAACCTTTCCAGTGTCATCAACC-3′. Real-time PCR reactions were carried out with 0.2 µl (0.15 µM) of each specific primers, 1 µl cDNA, 12.5 µl of the SYBR green master mix (Quanti Tech SYBR Green kit, Qiagen, Gmbh Hilden, Germany) and the final volume made up to 25 µl with RNase-free water. In the negative control cDNA was replaced by RNase free water. The reactions were performed on a DNA Engine Opticon 2 Continuous Fluorescence Detection System (MJ Research Inc., Waltham, MA). The programme used for real-time PCR was 3 min initial denaturation at 95°C, followed by 35 cycles of denaturation for 20 s at 95°C, annealing for 20 s (*PAL*: 53.7°C; *LOX*: 56.9°C; *AOC*: 56.5°C; *PR1*: 55.4°C; *PR2*: 51.5°C; *PR3*: 58°C; *Ubi3*: 51.5°C and extension for 20 s at 72°C. The fluorescence signal was measured immediately after incubation for 2 s at 75°C following the extension step, which eliminates possible primer dimer detection. At the end of the cycles, melting temperatures of the PCR products was determined between 65°C and 95°C. The specificity of amplicons was verified by melting curve analysis and agarose gel electrophoresis. Three biological replicates were independently carried out and three pots per treatment were set up for each biological replicate. Each leaf sample for RNA extract was collected from tomato leaves of the ‘receiver’ plant in each pot.

### Determination of AM colonization of tomato plants using nested PCR

In order to determine whether common mycorrhizal networks between ‘donor’ and ‘receiver’ plants were established through *Glomus mosseae*, a nested PCR, using universal eukaryotic primers for the first amplification and taxon-discriminating primers for the second, was performed on individual trypan blue-stained mycorrhizal root fragments of tomato.

#### DNA preparation

Individual trypan blue-stained mycorrhizal (treatment A, C and D) or nonmycorrhizal (treatment B) root fragments (1 cm long), or one spore of *G. mosseae*, were rinsed in sterile H_2_O, crushed in 40 µl of TE buffer (10 mM Tris-HCl, pH 8, 1 mM EDTA) and heated at 95°C for 10 min in the presence of 10 µl of 20% Chelex-100 (BioRad). The crude DNA suspension was separated from cellular fragments by centrifugation at 12000 ***g*** for 5 min and 5 µl of the suspension was used as soon as possible for the first amplification reaction.

#### PCR Primers

Nested PCR was performed to enhance the efficiency of the amplification in order to increase the amount of DNA available for cloning. Primers LR1 and NDL22 were designed from previously published alignments of the large ribosomal subunit [66] flanking the variable domains D1 and D2. The primer pair LR1 (5′-GCATATCAATAAGCGGAGGA-3′) and NDL22 (5′-TGGTCCGTGTTTCAAGACG-3′) [67] was used for the first amplification of DNA and the eukaryotic-specific primer combination 5.25 (5′-CCTTTTGAGCTCGGTCTCGTG-3′) and NDL22 for the second [68].

#### PCR amplifications

Primary polymerase chain reactions (PCR) were performed in a final volume of 20 µl containing 9.2 µl of water, 0.8 µl (10 µM) of each specific primers, 2 µl 10×PCR buffer containing 15mM MgCl_2_, 5 µl DNA, 2 µl 2.5mM dNTPs, 0.2 µl 5 U/µl *Taq* polymerase. Each reaction was overlayed with mineral oil and amplification was performed in a thermal cycler (MJ Research PTC-100) programmed as follows: initial denaturation cycle at 95°C (3 min), followed by 35 cycles of denaturation at 93°C (1 min), annealing at 58°C (1 min) and extension at 72°C (1 min); the last cycle was followed by a final extension at 72°C for 5 min. The amplification product obtained from mycorrhizal roots or spore DNA after the first PCR amplification with the primer pair LR1–NDL22 was 747 bp.

In the case of a nested PCR reaction, 5 µl of the first PCR amplification, diluted 1/1000, served as template for the second reaction and amplification conditions were as above except for 25 amplification cycles and an annealing temperature of 60°C. Products of the second PCR amplification were visualised and separated by electrophoresis in 1.2% agarose gels stained with ethidium bromide. Bands were subsequently cut, and amplified DNA was purified with the High Pure PCR Product purification kit (Roche Diagnostic GmbH, Mannheim, Germany). Sequencing was carried out by Beijing Genomics Institute using the NDL22 primers. Results were manually aligned using the program BIOEDIT Sequence Alignment Editor (http://www.mbio.ncsu.edu/BioEdit/bioedit.html) and the sequences were used to search the GenBank (accession no: EF554481) by the BLASTN program. The sizes of the amplification products were 367 bp (Fig S1). The sequence data belong to the sequences of *Glomus mosseae*, indicating the formation of CMNs through *G. mosseae* between ‘donor’ and ‘receiver’ tomato plants.

### Statistical analysis

SAS 8.0 (SAS Institute, Cary, North Carolina) package for Windows was used for statistical analysis. Bioassay data were obtained from four independent biological series with three pots per treatment. The data for enzymatic activities and gene expression levels were obtained from three independent biological series with three pots per treatment. For each treatment three replicates were maintained in a completely randomized design. All data were evaluated by two-way factorial analysis of variance (ANOVA) with treatment differences among means tested at *P* = 0.05 by Tukey post-hoc test.

## Supporting Information

Figure S1 Products of the second amplification of the nested PCR on DNA from *Glomus mosseae*-colonized tomato roots and spores using the primer pair 5.25-NDL22. Lanes 1–4 are nested PCR of roots of receiver plants from four treatments A, B, C and D, respectively. Lanes 6–9 are nested PCR of roots of donor plants from four treatments A, B, C and D, respectively. Lanes 5 and 10 show amplifications from two spores of *Glomus mosseae*. Four treatments included: A) a healthy tomato ‘receiver’ plant was connected with a neighboring *A. solani*-challenged tomato ‘donor’ plant inoculated with *G. mosseae*; B) a healthy ‘receiver’ plant was grown near *A. solani*-challenged ‘donor’ plant without mycorrhizal inoculation; C) a healthy mycorrhizal ‘receiver’ plant was grown near the pathogen-challenged mycorrhizal ‘donor’ plant but the two tomato plants separated by a water-proof membrane and D) a healthy ‘receiver’ plant was connected with the neighbouring ‘donor’ plant inoculated with *G. mosseae* without pathogen inoculation. DNA Markers were 100, 200, 300, 400, 500, 600, 700, 800, 900, 1000 and 1500 bp. The sizes of the amplification products were 367 bp.
(0.83 MB TIF)

Table S1 Results of ANOVA testing mycorrhizal infection rates, disease incidences and indices of tomato ‘receiver’ and ‘donor’ plants infected by *Alternaria solani*.
(0.08 MB DOC)

Table S2 Mycorrhizal infection rates, disease incidences and indices of tomato ‘receiver’ and ‘donor’ plants infected by *Alternaria solani* in four independent sets of experiments with three replicates/experiment for bioassays.
(0.08 MB DOC)

Table S3 Levels of six defence-related enzymes in leaves of tomato ‘receiver’ plants in response to common mycorrhizal networks (CMNs) connected with *Alternaria solani*-infected neighbouring tomato.
(0.08 MB DOC)
