# Supplementary material for: Interplant Communication of Tomato Plants through Underground Common Mycorrhizal Networks
Source: PLoS One. 2010 Oct 13;5(10):e13324. doi: 10.1371/journal.pone.0013324 (PMC2954164; doi:10.1371/journal.pone.0013324)
Supplement: Table S1 — Results of ANOVA testing mycorrhizal infection rates, disease incidences and indices of tomato ‘receiver’ and ‘donor’ plants infected by Alternaria solani. (0.08 MB DOC) [file pone.0013324.s002.doc]

**Table S1.** **Results of ANOVA testing mycorrhizal infection rates, disease incidences and indices of tomato ‘receiver’ and ‘donor’ plants infected by *Alternaria solani****.*

| Test items | Factors | SS | d.f. | MS | *F*-value | *P*-value |
| --- | --- | --- | --- | --- | --- | --- |
| Disease incidence of ‘receiver’ plants | Intercept | 114420.9 | 1 | 114420.9 | 1815.723 | 0.000000 |
|  | Experiment | 74.9 | 3 | 25.0 | 0.396 | 0.756520 |
|  | Treatment | 6297.3 | 3 | 2099.1 | 33.310 | 0.000000 |
|  | Experiment*Treatment | 1233.6 | 9 | 137.1 | 2.175 | 0.051367 |
|  | Error | 2016.5 | 32 | 63.0 |  |  |
| Disease index of ‘receiver’ plants | Intercept | 42326.07 | 1 | 42326.07 | 1114.687 | 0.000000 |
|  | Experiment | 136.90 | 3 | 45.63 | 1.202 | 0.324850 |
|  | Treatment | 5224.70 | 3 | 1741.57 | 45.865 | 0.000000 |
|  | Experiment*Treatment | 842.07 | 9 | 93.56 | 2.464 | 0.029177 |
|  | Error | 1215.08 | 32 | 37.97 |  |  |
| Disease incidence of ‘donor’ plants | Intercept | 93028.32 | 1 | 93028.32 | 1032.094 | 0.000000 |
|  | Experiment | 361.32 | 3 | 120.44 | 1.336 | 0.285994 |
|  | Treatment | 9312.83 | 2 | 4656.41 | 51.660 | 0.000000 |
|  | Experiment*Treatment | 1461.74 | 6 | 243.62 | 2.703 | 0.037782 |
|  | Error | 2163.25 | 24 | 90.14 |  |  |
| Disease index of ‘donor’ plants | Intercept | 35713.13 | 1 | 35713.13 | 1342.245 | 0.000000 |
|  | Experiment | 557.00 | 3 | 185.67 | 6.978 | 0.001545 |
|  | Treatment | 9810.67 | 2 | 4905.34 | 184.363 | 0.000000 |
|  | Experiment*Treatment | 1879.85 | 6 | 313.31 | 11.775 | 0.000004 |
|  | Error | 638.57 | 24 | 26.61 |  |  |
| Mycorrhizal infection rates of ‘receiver’ plants | Intercept | 114420.9 | 1 | 114420.9 | 1815.723 | 0.000000 |
|  | Experiment | 74.9 | 3 | 25.0 | 0.396 | 0.756520 |
|  | Treatment | 6297.3 | 3 | 2099.1 | 33.310 | 0.000000 |
|  | Experiment*Treatment | 1233.6 | 9 | 137.1 | 2.175 | 0.051367 |
|  | Error | 2016.5 | 32 | 63.0 |  |  |
| Mycorrhizal infection rates of ‘donor’ plants | Intercept | 125676.1 | 1 | 125676.1 | 4414.817 | 0.000000 |
|  | Experiment | 45186.4 | 3 | 15062.1 | 529.111 | 0.000000 |
|  | Treatment | 79.2 | 3 | 26.4 | 0.927 | 0.432717 |
|  | Experiment*Treatment | 354.9 | 9 | 39.4 | 1.385 | 0.213461 |
|  | Error | 1821.9 | 64 | 28.5 |  |  |

Four independent sets of experiments with 3 replicates/experiment were conducted for bioassays. Four treatments included: **A**) a healthy tomato **‘**receiver**’** plant was connected with a neighboring *A. solani-*challenged tomato **‘**donor**’** plant through CMNs; **B**) a healthy **‘**receiver**’** plant was grown near *A. solani*-challenged **‘**donor**’** plant but no mycorrhiza was applied; **C**) a healthy mycorrhizal **‘**receiver**’** plant was grown near the pathogen-challenged mycorrhizal **‘**donor**’** plant but the two tomato plants separated by a water-proof membrane and **D**) a healthy **‘**receiver**’** plant was connected with the neighbouring plant by CMN without pathogen inoculation.
