# Supplementary material for: Interplant Communication of Tomato Plants through Underground Common Mycorrhizal Networks
Source: PLoS One. 2010 Oct 13;5(10):e13324. doi: 10.1371/journal.pone.0013324 (PMC2954164; doi:10.1371/journal.pone.0013324)
Supplement: Table S2 — Mycorrhizal infection rates, disease incidences and indices of tomato ‘receiver’ and ‘donor’ plants infected by Alternaria solani in four independent sets of experiments with three replicates/experiment for bioassays. (0.08 MB DOC) [file pone.0013324.s003.doc]

**Table S2. Mycorrhizal infection rates, disease incidences and indices of tomato ‘receiver’ and ‘donor’ plants infected by *Alternaria solani* in four independent sets of experiments with three replicates/experiment for bioassays.**

| Test items | Treatment | Experiment 1 | Experiment 2 | Experiment 3 | Experiment 4 |
| --- | --- | --- | --- | --- | --- |
| Disease incidence (%) of ‘receiver’ plants | A  B  C | 38.5±1.3b | 20.6±4.0b | 30.6±0.5b | 36.1±1.4a |
|  |  | 62.7±4.2a | 68.7±3.2a | 67±4.1a | 54.9±5.9a |
|  |  | 42.5±5.1b | 56.2±9.6a | 49.5±7.5ab | 44.3±3.0a |
|  | D | 48.8±4.6ab | 52.2±3.6a | 54.9±2.5a | 53.7±9.9a |
| Disease index (%) of ‘receiver’ plants | A  B | 18.5±1.0c | 11.9±1.2c | 18.6±1.7c | 17.2±3.1a |
|  |  | 37.5±0.4a | 59±3.8a | 44.8±1.3a | 41.7±1.7a |
|  | C | 23.8±3.4bc | 29.5±4.9b | 29.1±1.7b | 31.6±9.2a |
|  | D | 28.8±1.6ab | 27±0.5b | 28.8±2.9b | 27.3±5.9a |
| Disease incidence (%) of ‘donor’ plants | A  B | 40.8±2.0b | 23.7±9.2b | 27.0±3.5b | 40.1±5.3b |
|  |  | 70.0±5.8a | 84.2±2.7a | 70.5±5.8a | 63.0±4.2a |
|  | C | 57.5±4.9ab | 42.1±7.2b | 45.5±7.0b | 45.7±3.6ab |
|  | D | 0c | 0c | 0c | 0c |
| Disease index (%) of ‘donor’ plants | A  B | 16.3±0.2c | 13.5±4.3b | 11.6±0.8c | 17.2±0.8c |
|  |  | 46.1±4.3a | 77.8±6.0a | 47.2±2.4a | 44.5±2.6a |
|  | C | 27.0±0.6b | 22.9±4.3b | 24.1±0.4b | 29.7±1.2b |
|  | D | 0d | 0c | 0d | 0d |
| Mycorrhizal infection rates (%) of ‘receiver’ plants | A  B  C | 42.2±5.2a | 39.4±2.1b | 30.0±4.4b | 33.2±8.8b |
|  |  | 0b | 0c | 0c | 0c |
|  |  | 48.6±1.4a | 49.0±0.7a | 49.2±1.4a | 48.0±0.6a |
|  | D | 39.6±1.9a | 38.8±1.7b | 33.8±1.6b | 32.2±2.2b |
| Mycorrhizal infection rates (%) of ‘donor’ plants | A  B  C | 54.0±1.1b | 54.2±2.7a | 52.8±4.5ab | 61.0±2.7a |
|  |  | 0d | 0c | 0c | 0c |
|  |  | 41.6±0.5c | 40.9±1.5b | 45.0±1.3b | 43.5±2.8b |
|  | D | 65.0±3.4a | 59.8±4.8a | 56.8±2.3a | 59.6±1.9a |

Four treatments included: **A**) a healthy tomato **‘**receiver**’** plant was connected with a neighboring *A. solani-*challenged tomato **‘**donor**’** plant through CMNs; **B**) a healthy **‘**receiver**’** plant was grown near *A. solani*-challenged **‘**donor**’** plant but no mycorrhiza was applied; **C**) a healthy mycorrhizal **‘**receiver**’** plant was grown near the pathogen-challenged mycorrhizal **‘**donor**’** plant but the two tomato plants separated by a water-proof membrane and **D**) a healthy **‘**receiver**’** plant was connected with the neighbouring plant by CMN without pathogen inoculation. Values are means ± standard error from three replicates in the same experiment. Significant differences (*P*<0.05 using Tukey post-hoc test) among treatments are indicated by different letters.
