# Supplementary material for: Interplant Communication of Tomato Plants through Underground Common Mycorrhizal Networks
Source: PLoS One. 2010 Oct 13;5(10):e13324. doi: 10.1371/journal.pone.0013324 (PMC2954164; doi:10.1371/journal.pone.0013324)
Supplement: Table S3 — Levels of six defence-related enzymes in leaves of tomato ‘receiver’ plants in response to common mycorrhizal networks (CMNs) connected with Alternaria solani-infected neighbouring tomato. (0.08 MB DOC) [file pone.0013324.s004.doc]

***Table S3. Levels of six defence-related enzymes in leaves of tomato ‘receiver’ plants in response to common mycorrhizal networks (CMNs) connected with Alternaria solani-infected neighbouring tomato***.

| Hours after pathogen inoculation | Treatment | Enzymatic activity (U h^-1^ g^-1^ FW) | | | | | |
| --- | --- | --- | --- | --- | --- | --- | --- |
|  |  | POD | PPO | Chitinase | β-1,3-Glucanse | PAL | LOX |
| 0 | A | 249.5±15.3a | 53.2±7.7a | 34.1±1.7a | 55.4±0.8a | 0.48±0.02a | 3.3±0.7a |
|  | B | 264.7±51.0a | 66.4±7.7a | 31.3±4.1a | 59.1±2.3a | 0.40±0.02a | 3.4±1.0a |
|  | C | 242.4±36.3a | 57.4±11.8a | 33.6±2.0a | 54.8±4.5a | 0.42±0.10a | 2.7±0.7a |
|  | D | 254.9±9.6a | 66.5±15.3a | 34.7±6.5a | 53.1±1.1a | 0.46±0.08a | 3.6±1.1a |
|  |  |  |  |  |  |  |  |
| 18 | A | 696.7±56.4a | 138.4±26.4a | 50.5±2.8a | 64.4±5.2a | 0.90±0.14ab | 3.87±0.59a |
|  | B | 363.0±45.8b | 105.4±17.2b | 42.5±3.8ab | 50.9±4.4b | 0.72±0.10ab | 2.29±0.34a |
|  | C | 473.3±64.0b | 150.8±20.0a | 42.8±5.2ab | 55.3±5.9ab | 0.94±0.13a | 4.01±1.01a |
|  | D | 438.5±48.3b | 108.6±16.3b | 36.7±6.7b | 45.7±3.8b | 0.66±0.09b | 3.11±0.45a |
|  |  |  |  |  |  |  |  |
| 65 | A | 827.8±66.0a | 125.1±20.5a | 37.0±3.0a | 58.6±4.6a | 1.21±0.08a | 3.43±0.22a |
|  | B | 457.4±51.4b | 95.4±16.5b | 24.4±3.4b | 42.8±2.4b | 0.42±0.03c | 1.84±0.24b |
|  | C | 475.6±77.9b | 106.3±18.1b | 29.0±2.9b | 43.7±3.8b | 0.70±0.03b | 2.32±0.18b |
|  | D | 371.9±67.2b | 102.9±20.0b | 29.2±3.7b | 49.0±2.3b | 0.57±0.07bc | 1.89±0.39b |
|  |  |  |  |  |  |  |  |
| 100 | A | 733.3±42.2a | 124.1±17.7a | 40.8±3.6a | 77.1±11.3a | 1.40±0.27a | 4.39±0.23a |
|  | B | 322.2±34.3c | 73.8±6.9b | 27.6±2.4b | 48.4±2.6b | 0.76±0.07b | 2.61±0.29b |
|  | C | 485.2±23.9b | 82.1±10.8b | 30.6±5.4b | 53.6±2.5b | 0.77±0.09b | 3.28±0.37b |
|  | D | 365.2±46.7c | 77.6±7.3b | 28.3±4.4b | 52.8±2.0b | 0.50±0.04c | 2.73±0.29b |
|  |  |  |  |  |  |  |  |
| 140 | A | 935.9±81.1a | 164.3±11.6a | 38.0±2.3a | 80.0±6.4a | 0.83±0.12a | 5.19±0.37a |
|  | B | 448.5±29.1c | 106.8±9.0b | 25.1±4.6b | 45.9±2.8b | 0.44±0.07b | 3.08±0.54b |
|  | C | 647.0±83.8b | 102.6±7.9b | 25.2±2.4b | 56.1±4.6b | 0.50±0.10b | 3.72±0.53b |
|  | D | 528.1±61.1bc | 101.0±12.5b | 27.9±2.7b | 53.4±2.8b | 0.55±0.08b | 3.48±0.51b |

Four treatments included: **A**) a healthy tomato **‘**receiver**’** plant was connected with a neighboring *A. solani-*challenged tomato **‘**donor**’** plant through CMNs; **B**) a healthy **‘**receiver**’** plant was grown near *A. solani*-challenged **‘**donor**’** plant but no mycorrhiza was applied; **C**) a healthy mycorrhizal **‘**receiver**’** plant was grown near the pathogen-challenged mycorrhizal **‘**donor**’** plant but the two tomato plants separated by a water-proof membrane and **D**) a healthy **‘**receiver**’** plant was connected with the neighbouring plant by CMN without pathogen inoculation. Three sets of experiments were independently carried out and three pots per treatment were set up for each set of experiments. Values are means ± standard error. Significant differences (*P*<0.05 using Tukey post-hoc test) among treatments at the same time point are indicated by different letters.
